# How to Build and How not to Build an Implicit Measure in Behavior Analysis: A case Study Using the Function Acquisition Speed Test

**DOI:** 10.1007/s40614-023-00387-w

**Published:** 2023-08-11

**Authors:** Aideen Watters, Jamie Cummins, Bryan Roche

**Affiliations:** 1 Maynooth University, Kildare, Ireland; 2 Ghent University, Gent, Belgium

**Keywords:** Implicit association test, Function acquisition speed test, Implicit relational assessment procedure, Stimulus equivalence, Functional response classes

## Abstract

This article provides a comprehensive overview of the development of a behavior-analytic alternative to the popular implicit association test (IAT), namely, the function acquisition speed test (FAST). The IAT appears, *prima facia*, to indirectly assess participants’ learning histories with regard to the categorization of stimuli. However, its origin within cognitive psychology has rendered it replete with mentalism, conceptual ambiguity, statistical arbitrariness, and confounding procedural artifacts. The most popular behavioral alternative to the IAT, the widely used implicit relational assessment procedure (IRAP), has inherited many of these concerning artifacts. In this article, we present a behavior-analytic critique of both the IAT and IRAP, and argue that a behavior-analytic approach to implicit measures must have *stimulus control* front and center in its analysis. We then outline a series of early research studies that provided the basis for a potentially superior procedure within our field. We go on to outline how this early research was harnessed in stepwise research, guided by a strict adherence to traditional behavior-analytic methods for the analysis of stimulus relations, to increasingly modify a test format fit for the behavior analyst interested in assessing stimulus relatedness.

In 1998, Greenwald and Banaji first introduced their implicit association test (IAT), promising an indirect and discrete measure of “unconscious bias” or “mental associations.” This single test has made an enormous impact on the field of social psychology and on psychology in general. The Greenwald et al. (1998) article has been cited more than 15,000 times and the IAT has been used in hundreds of studies attempting to measure implicit attitudes (e.g., ethnic/racial discrimination: Oswald et al., 2013; gender: Hansen et al., 2019; racial preference: Dasgupta et al., 2000; self-biases: Nosek et al., 2002; voting intention: Greenwald et al., 2009).

The idea that unconscious cognitive events and mental representations could be measured by a simple test intrigued psychologists, to say the least. Although behavior analysts might be initially skeptical of such claims and take issue with the use of mentalistic terms, procedures such as the IAT can of course also be subject to conceptual analysis from a behavior-analytic perspective. In fact, the development of such “implicit measures” directly parallels developments of similar procedures within our field for different but related purposes.

In this article, we outline the basic methodology of the IAT, and illustrate its conception as measuring the strength of “mental associations,” and from this inferring “unconscious biases” and “attitudes.” We then raise some generic concerns regarding the conceptualization and methodology of this test and, related to this, the implicit relational assessment procedure (IRAP; Barnes-Holmes et al., 2010a). We then focus on the development of the FAST methodology as an excellent case study in how to build an implicit measure using a bottom-up, cumulative approach. As we will argue, the FAST represents an improvement upon both the IAT and IRAP because there has been a more concentrated effort to understand the functional properties of the measure, as well as being more methodologically sound and involving less opaque statistical abstraction in the scoring method. Unlike the IRAP, the FAST has been unambiguously presented *ab initia* solely as a measure of the relatedness of stimuli, with a functional understanding of the task build up across a series of ground-up empirical research studies. It should be noted, however, that it may be cautiously speculated that stimulus relatedness might be used as a proxy for “attitudes,” depending on how these are functionally defined (see O’Reilly et al., 2015).

## The Implicit Association Test

The IAT is a computer-based test designed to assess “mental associations” thought to underlie implicit biases or attitudes. Derived from a connectionist perspective on cognition, the creators of the test conceived an “attitude” as the probability that the activation of a mental concept (e.g., the mental concept of a particular racial group) will lead to the activation of a valence attribute mental concept (e.g., positive valence; Greenwald et al., 2002). The “implicit” aspect of the test derives from the idea that the measure captured the activation of these concepts in an unconscious manner. The developers of the test advocated that such implicit attitudes may then have a downstream impact on behavior in a similarly unconscious manner. From such a perspective, implicit bias is viewed in essence as a causal latent variable; a mental structure in the form of associations that affects upon behavior in an unconscious manner (De Houwer, 2019). The IAT was partly developed in response to the problem of presentation bias in the measurement of attitudes and cognitive biases (see Goffman, 2002; Greenwald & Breckler, 1985). Although its developers did not assume that implicit biases were any more “authentic” than self-presentational biases, automatic responses were nevertheless of research interest, especially in sensitive research contexts in which automatic biases were unlikely to occur unmasked (Greenwald & Banaji, 1995).

In the IAT, participants are presented with individual stimuli representing one of two distinct categories of target stimuli (e.g., flowers or insects), or one of two categories of attribute stimuli (e.g., “good” and “bad”). Stimuli representing each of these four categories are presented individually on separate trials on a computer screen. The critical task blocks are preceded and intermixed with a series of practice blocks involving the same stimuli. During the critical blocks of the test, a participant is instructed to press a left or right keyboard button (e.g. left: “E” key, right: “I” key) on each trial. The specific response requirements are outlined in rules presented at the beginning of each block, which remain present at the top of the computer screen during the block (e.g., “press left for names of flowers and good words, press right for names of insects and bad words”). Each critical block of the test (i.e., “consistent” and “inconsistent”) typically contains 60 trials, divided into a first block of 20 trials and a second block of 40 trials each. In the consistent block, required response configurations are assumed to be consistent with the associations between mental concepts of participants (e.g., flowers and good words share a response, insects and negative words share another response). In the inconsistent block, the response configurations are assumed to be inconsistent with these associations. Relatively faster responding during one block compared to the other is assumed to reflect preexisting associations between mental concepts.

Despite enormous interest (or more likely, because of it), the IAT has also been the subject of considerable conceptual and methodological critique. In the following section we will consider the most prominent of these critiques, focusing specifically on those that would be of most interest and relevance to a behavior-analytic audience.

### The IAT and its Curious Methodology

The IAT is premised upon several assumptions. Some of these assumptions are testable, but some represent *a priori* mentalistic assumptions and explanations of behavior which are situated at a different level of analysis than the behavior-analytic approach. For instance, researchers have critiqued the assumption that attitudes are best understood as associations between mental concepts (see Hughes et al., 2011). Its developers suggest that the presentation of a stimulus in the task *activates* a mental representation of another related stimulus, and that this activated association affects tasks performance. Notably, treatments of the IAT frequently conflate the behavioral effect observed in the task (i.e., response time differences between blocks) with this proposed mental mechanistic explanation, although others (e.g., De Houwer et al., 2013) have more recently argued that this conflation often confounds the interpretation of IAT scores by virtue of failing to adequately separate between observed effects in the measure (e.g., an IAT score) and corresponding explanatory accounts (e.g., association activation).

The behavior analyst would of course object to the treatment of mental events as viable independent variables. If some procedural features of the IAT have been developed as a function of the mentalistic assumptions which the behavior analyst would reject, then it follows that behavior analysts should exercise caution in their own measures inheriting these features.

One rather elementary matter that has not been addressed by the IAT is the issue of how test scores *functionally* relate to known magnitudes of the construct of interest. In other words, we do not know how scores on the measures provide an adequate index of the various constructs they claim to measure. Social cognitive researchers have traditionally approached the issue of the relationship between implicit test scores and variances in the various constructs of interest in terms of construct validity based on test theory (e.g., Schimmack, 2019). That is, they assume that underlying the test score exists a mental construct or mechanism (such as unconscious beliefs or biases), which can be more or less accurately indexed by this test score. The reliability of this index has traditionally been examined on the basis of explicit–implicit measure correlations and estimates of incremental validity over those provided by explicit measures alone. It is interesting that after 2 decades of such research, evidence for sound incremental validity over explicit measures is actually relatively weak (Meissner et al., 2019).

As a result of the foregoing problem, critics have specified particular processes that compromise the reliability of IAT scores as a direct index of the strength of mental associations. For example, Calanchini et al. (2014) identified nonattitudinal processes influencing IAT scores. In particular, they found that detection ability (i.e., the ability to discriminate target stimuli according to the active response contingencies) influences IAT performances and therefore scores. That is, the ability to discriminate the correct response requirement on each task affects IAT response times, irrespective of “attitudes” toward the target concept. Other researchers (e.g., Meissner et al., 2019) have suggested that (*inter alia*) the convergent validity of the IAT has been compromised by a focus on the measurement of mental *associations*, and that research using these measures might benefit from instead focusing on *propositions* (see also De Houwer et al., 2015).

Despite having proposed multiple means by which the validity of the IAT can be enhanced, few include a reliance on the study of laboratory-controlled phenomena that can be objectively quantified as the basis of the test metrics (see Cummins & De Houwer, 2022, for an exception). In addition, researchers continue to use a common-sensical convergent validity approach to assessing the validity of their implicit measures (e.g., Thomton & Antkin, 2020), even within behavior analysis (e.g., Barnes-Holmes et al., 2010b; Barnes-Holmes et al., 2009; Cabrera et al., 2021; Chan et al., 2009; Kelly & Barnes-Holmes, 2013; McKenna et al., 2016; Perez et al., 2019).

The behavior analyst should not be satisfied with such a correlational approach to validity for the simple reason that the explicit measures employed in the process are themselves often open to question regarding validity (e.g., questionnaires, Likert scales). In effect, it is important to understand that from a behavior-analytic perspective, simply identifying correlations across measures at the group level does not satisfy the requirement for the demonstration of a functional relationship between measures, particularly in a context in which neither of the measures is functionally understood from the bottom-up under controlled laboratory conditions. In contrast, a behavior-analytic approach might involve systematic experimental manipulations that produce analogs of the performance observed in implicit measures as a solid foundation upon which to build a functional understanding of those performances. Within that approach, the issue of inferring the validity of novel measures based upon statistical convergence with other measures is circumvented entirely, insofar as the measure would be understood first and foremost in terms of basic principles, rather than in terms of constructs or hypothetical processes. In addition, such a strategy would provide a better understanding of individual-level processes at work in each performance, something highly valued by the behavior-analytic community. In other words: a behavior-analytic approach to implicit measures, much like behavior-analytic approaches to any other subject matter, must put *stimulus control* at the center of its analysis and development.

Several other critiques of the IAT have been offered by IAT researchers, such as those relating to confounds in stimulus exemplar selection (e.g., De Houwer, 2002), susceptibility to conscious control (Fiedler et al., 2006), and cognitive ability (Klauer et al., 2010), to name but a few. Behavior analysts have also raised concerns about the IAT, as well as having attempted to provide functional-analytic accounts of the IAT’s core process based on both behavior theory and several empirical studies. One such criticism that has been raised in several articles (e.g., Cartwright et al., 2016) relates to the curious response feedback procedure employed. In particular, although the putative role of feedback in the IAT is to facilitate more accurate performance by the participant, it is surprising that only negative feedback is provided following errors, whereas positive feedback is not provided at any stage. In particular, incorrect responses are consequated by the presentation of a red X on screen and a requirement to produce the alternate correct response to terminate the trial. This is an extremely inefficient way to teach. That is, in a context in which there is no competing behavior requiring extinction, there is rarely a need to consequate responses by aversive stimuli. A century of research has shown that not only is positive reinforcement the procedure of choice for ensuring efficient learning, but that the negative side effects of aversive consequences for responding are a considerable confound during the acquisition of behaviors (see Axeirod & Apsche, 1983; Iawata, 1987). The threat of aversive consequences leads to avoidance behaviors that competes with the acquisition of approach behaviors (see Iawata, 1987, for a review of these complex issues). It would seem to be a reasonable suggestion that the red X used during response feedback in the IAT may function as a conditioned aversive punisher, which is presented intermittently across trial blocks, thus potentially interfering with the acquisition of fluent responding under tight stimulus control. The presentation of such intermittent aversive stimuli is a well-understood impediment to smooth acquisition during learning (e.g., Church & Raymond, 1967). In other words, a method of teaching that relies solely on S- rather than S+ control is a suboptimal method.

Evidence that the use of negative feedback interferes with response class acquisition was generated by social cognitive researchers themselves before the advent of the IAT in the context of research into the Stroop task (Stroop, 1935). In particular, Rabbit and Rodgers (1977) found significant decreases in response fluency following negative feedback during Stroop tasks that were sufficiently statistically unrelated to the task itself that they suggested the omission of response time recordings for all Stroop trials following errors. Retaining response times for trials subsequent to the presentation of negative feedback has the consequence that if one block of the IAT is already somewhat more difficult than another (i.e., producing slightly longer response times), then negative feedback may serve to further enhance the response time differences across blocks. However, it is unclear to what extent this may occur. The use of imbalanced feedback methods may exaggerate response time differentials across blocks in the direction of the hypothesis, for instance. This represents an example of precisely how *not to* establish effective stimulus control over accurate responding.

A further aspect of the IAT feedback procedure also exaggerates recorded response times for errors in line with hypotheses. The method has been discussed openly in the social cognitive literature (e.g., Greenwald et al., 2003), but aside from under-the-radar commentary by the developers of the FAST procedure (e.g., O’Reilly et al., 2012; Ridgeway et al., 2010), it has curiously not raised an eyebrow within behavior analysis, let alone led to open controversy. In particular, the IAT does not in fact record response times from the point of stimulus presentation to the emission of a response, but from the point of stimulus presentation to the production of a *correct response*. The time taken to correct an error response following the presentation of the red X, is roughly 400 ms (see Greenwald et al., 2003). Thus, response latencies on all error trials are enhanced by approximately 400 ms, thereby exaggerating the response time differential across blocks in the direction of the hypothesis. This method translates error rate differences across blocks into response time differentials in an opaque and conceptually questionable manner. Greenwald et al. (2003) outlined how this 400 ms additional “built-in” time penalty was sufficient to secure reliable and stronger IAT effects and should be retained as part of the standard procedure in preference to a previous and almost incomprehensible practice of manually recording response times for error responses as the average response time for the whole trial block + 600 ms. This penalty had been previously chosen on the basis that it appeared to be necessary to produce reliable effects based on response times in the IAT. This effort to convert response accuracy into response latency using arbitrary time penalties seems conceptually questionable, and at minimum it is not clear why these specific latencies ought to be chosen. Indeed, some users of the IAT have opted for improved methods that utilize advanced modelling techniques to account for these speed-accuracy trade-offs in a more nuanced manner (e.g., Röhner & Lai, 2021). This general strategy is at odds with a behavior-analytic philosophy and is a poor substitute for a thorough understanding of stimulus control within the task.

It is interesting that according to the findings of Greenwald et al. (2003), the *D* score is relatively unaffected by the exclusion of the corrective feedback procedure (i.e., by substituting it with a larger response time penalty than achieved using the response correction procedure). This raises questions about the precise function of response feedback in the IAT. That is, if it has no effect on the speed of responding, then why is the procedure employed? One reason may be that its retention is required for its function in enhancing response times due to these being recorded from the time of stimulus onset to the production of a correct response following errors and negative feedback. However, one study found that speed of responding is in fact improved by the presence of the feedback (e.g., Ellithorpe et al., 2015). In contrast, Richetin et al. (2015) found that the absence of feedback (but imposition of the larger manual time penalty) makes little difference to the reliability or validity of the *D* score. It is important to note, however, those researchers found that the combination of the larger response time penalty for error responses *combined* with the inclusion of the response correction procedure led to instability in the validity and reliability of the resulting *D* score. This finding suggests that the red X following error responses is indeed leading to response instability, but to an extent that is only significant when the effects are combined with those of larger arbitrary response time penalties.

One recent study specifically addressed an unfortunate corollary of an imbalanced feedback procedure. In particular, Ellithorpe et al. (2015) suggested that the use of negative feedback only is likely to have spurious effects on performances insofar as it may function indirectly to decrease the fluency of one response pattern, or increase the orthogonal one, with little control over the outcome in either case. For example, they suggest the hypothetical scenario in which negative feedback on tasks during which African American and positive evaluative stimuli share a response may indirectly increase responses associating African American stimuli with negative stimuli. This could actually interfere with the very stimulus association under assessment in the task (see also Epifania et al., 2023; Hussey & De Houwer, 2019; Olson & Fazio, 2004; Olson et al., 2009). The use of a more balanced feedback procedure (in which both correct and incorrect responses are consequated by feedback) would help to address this problem by tightening stimulus control over the desired response repertoire.

It is worth highlighting at this point that many of the foregoing issues arise only because of the commitment in social cognition research to the response time-based index of performance on the IAT. In contrast, within the behavioral tradition, stimulus control is usually established in the first instance through the use of accuracy measures, with response time being indexed only as an adjunct measure once accuracy has been maximized (see Binder, 1996). For instance, the effectiveness of training intended to lead to the emergence of derived stimulus equivalence relations is usually assessed using an accuracy criterion alone (e.g., Fields et al., 1990), although response times have occasionally been used as an auxiliary metric (see Fields et al., 2014). Thus, given that the fluencies of the various response repertoires being measured in the IAT are unknown except for how they are indexed by the IAT itself, it would be more prudent to use response accuracy as a measure of stimulus compatibility until those very compatibilities have been established in principle in the first instance. Indeed, using accuracy as the primary metric in implicit measures like the IAT appears to offer advantages beyond response times (cf. Cummins & De Houwer, 2022). To use response speed alone as the index of the presence or absence of relations between stimuli, whose degree of relatedness is otherwise unknown, is conceptually questionable at a minimum. Although behavior analysts have no fundamental objection to the use of response times in such research contexts, we are aware that response times are typically not normally distributed (see Whelan, 2008) and that the application of algorithms (such as the IAT-*D* score) are subsequently required to normalize data distributions, resulting in scores that are highly abstracted from the behavior of interest. An alternative is to employ sophisticated statistical methods designed for the analysis of nonparametric data, but such strategies do not address the fundamental issue of a lack of stimulus control within the measurement procedure.

The persistence in employing response latency measures within the IAT (and IRAP; see below) brings with it even more unfortunate requirements to deal with the fallout from this noisy measure in ways that make the resulting test score even more opaque than we have outlined thus far. In particular, the widely used IAT-*D* score algorithm (Greenwald et al., 2003) requires the use of response time truncation, recoding, and data elimination methodologies intended to normalize data and maximize the chances of statistically significant test effects across a number of domains based on common analyses done using the IAT (see Greenwald et al., 2003). In particular, the IAT score calculation process first involves the removal of all response times above 10,000 ms, removal of all participant data where > 10% response times are above 10,000 ms or below 300 ms, the recoding of all response times between 3,000 ms and 10,000 ms to 3,000 ms and recoding of all response times < 300 ms to 300 ms. A mean response time is then calculated for each test block following these adjustments. The difference in response latency between two critical consistent and two critical inconsistent blocks is calculated and divided by the standard deviation of the response latencies across the critical blocks combined, resulting in the IAT-*D* score. Of course, there is no *a priori* basis for objection to the use of scoring algorithms. However, it is critical to note that complex scoring algorithms are employed at the expense of interpretability; a D score cannot provide immediate and direct insights into the behavior that produced it due to these extensive data transformations. Once again, a more desirable approach would be to improve the stimulus control exerted over behavior within the task itself.

## The Implicit Relational Association Procedure

Although the current article was not intended *primarily* as either an exploration or a critique of the IAT or the IRAP, a brief outline and critique of both is central to appreciating the benefits of the FAST methodology we aim to describe. If we are to describe (what we consider to be) the right steps to take in developing a behavior-analytic implicit measure, the IRAP sets the stage for us to describe (what we consider to be) the wrong steps in such an enterprise. For this reason, we will now briefly outline the IRAP procedure as a methodology that arose out of the research literature into stimulus equivalence, and from relational frame theory (Hayes et al., 2001; see Power et al., 2009).

Like the IAT, the IRAP is a computer-based task that records reaction times across several practice and test blocks. However, the IRAP assesses multiple stimulus relations rather than mere equivalence relations or “associations” between stimuli. That is, it is interested in assessing the nature of two different relations between categorical stimuli of interest and (usually) evaluative stimuli. During a critical trial, participants are presented with a category stimulus at the top of the screen, beneath which an attribute stimulus is visible. Instructions at the bottom left and right corners of the screen indicate the keyboard operanda and the response requirement on that trial. Relational cues (e.g., More or Less) or relational coherence indicators (e.g., True or False) present on the screen are the choice (or comparison) stimuli to which the participant responds on the keyboard. For example, in a gender bias IRAP, male and female class exemplars label might be employed as category stimuli, whereas positive and negative evaluative terms might be employed as attribute stimuli. Because this test format expands upon the IAT by assessing both compatibilities and incompatibilities between stimulus classes according to any relations of interest (e.g., comparison), four key trial blocks are required rather than just two. Across the four trial types, participants are required to respond by confirming or disconfirming the compatibility between two on-screen stimuli in terms of a particular relation specified in the continuously available on-screen instructions. For example, the stimulus combinations Male-Positive, Male-Negative Female-Positive, Female-Negative might be presented across separate trials in the presence of two different possible sets of response instructions. One set of instructions might require the participant to confirm that male stimuli and positive stimuli are related by pressing the True button, whereas another might require them to respond as if male and positive stimuli are not equivalent by pressing the False button.

Because the IRAP presents twice the number of task types as the IAT, it is a very complex, time consuming, and difficult task for participants to complete and thus suffers with a high attrition rate due to its requirement for participants to achieve typically > 80% accurate responding and a median response latency of < 2000 ms within the practice blocks presented prior to the critical test blocks. This results in attrition rates well in excess of those typically observed in behavioral studies, ranging from about 20% exclusion (e.g., Geist et al., 2023) to over 50% (e.g., Errasti et al., (2019). These attrition rates are not a mere inconvenience. They represent the loss of data that would allow researchers to study the very phenomenon of interest in the measure and belie a focus on something other than the experimental analysis of behavior. That is, a failure to reach a high level of steady state fluent behavior on a particular trial type during a practice block could be the result of the very resistance to change in the formation of novel relations that should be studied by the measure itself. In other words, resistances to change in the formation of relations trained within the IRAP should be visible, albeit with some noise, even within the practice blocks. Publishing data on whether or not this is the case and the degree to which IRAP effects are visible from the very first trial on each task block would allow the research community to assess the function of the practice blocks themselves in stabilizing response time differences across the task types, presumably for purposes of clarity of effects and statistical inference. It would also allow the research community to decide whether or not the metric should in fact focus on the noisy acquisition of relations trained in the IRAP during the practice blocks themselves as the primary and most transparent measure of the phenomenon of interest, with improvements in stimulus control, rather than the elimination of noisy data, as the means by which the effect can be captured by the relevant metric. Such an approach would be fundamentally at odds with the modal usage of the IRAP, however, and a complete overhaul of the scoring and method of the IRAP appears unlikely.

Although the IRAP possesses some methodological and theoretical distinctions from the IAT, it also bears some striking similarities in terms of the use of practice blocks, its commitment to response time over accuracy measures, its scoring algorithm with the same data recoding and exclusion criteria (the *D*-IRAP), use of prior instructions rather than a shaping procedure for task performance, the continuous presentation of instructions on screen during trials, the positioning of instructions on screen, the use of the red X as response feedback on error trials only, the recording of response times from stimulus presentation to first correct response to build in an artificial time penalty for errors, the reliance on instructions rather than response windows to ensure rapid responding, and even the use of identical operanda on the computer keyboard (i.e., the E and I keys rather than the Z and M keys as used traditionally within the study of derived stimulus relations).

An important point in the context of the current article, however, is that the test has been developed almost entirely in the absence of laboratory-controlled experimentation of the type typically considered necessary for the development of behavior-analytic methodologies. From the first study onwards, the test format has barely evolved in terms of demonstrated improvements in stimulus control and explications of its utility have almost exclusively involved the use of real-world uncontrolled stimulus classes. As a result, the validity and conceptual meaning of scores in the measure can only be surmised through convergence with other measures (which, as discussed above, is problematic). With almost no exception (cf. Hussey et al., 2016a) this has been the mode of evidence used to support the IRAP’s validity. Although welcome. those few studies that have attempted to develop a functional understanding of performance on the IRAP, have focused on procedural features such as the nature of the rule-based instructions provided at the outset of the task (e.g., Finn et al., 2016), rather than on explicating core behavioral processes through experimental analogs of test effects using laboratory-controlled stimulus classes. In the absence of ground-up research relating IRAP scores to known independent measures of stimulus relatedness, it is almost impossible for us to determine the meaning, reliability, and validity of IRAP scores.

Perhaps some of the foregoing issues might have been addressed in research to date had the purpose of the procedure been more clearly linked to known behavioral processes and demonstrated in the laboratory in basic research. This was unlikely to have occurred, however, because of opaqueness about precisely what the test was intended to measure, as addressed in a recent article attempting to clarify the original purpose as that of measuring stimulus relations (Barnes-Holmes & Harte, 2022). In that same article, the claim is made that any perceived ambiguity about the purpose of the test as anything other than to measure stimulus relations occurred as a result of misunderstandings within the wider research community. However, given the titles of dozens of articles produced by the original creators of the test, and the name of the test itself, such a claim might be fairly described as revisionist (see Hussey, 2022). In particular, the constructs claimed to be measured across IRAP studies including the creator of the test as co-author include, but are not limited to *implicit beliefs* (e.g., Barnes-Holmes et al., 2006; Dawson et al., 2009), *attitudes* (e.g., Cullen et al., 2009; McKenna et al., 2016; Roddy et al., 2009), *cognition* (e.g., Reume, De Houwer, & Barnes-Holmes, 2013), *stereotyping* (e.g., Barnes-Holmes, Murphy et al., 2010a; Power et al., 2017), *group favoritism* (e.g., Hughes et al., 2017), *verbal relations* (e.g., Barnes-Holmes et al., 2008), *self-esteem* (e.g., Vahey et al., 2009), *fear* (e.g., Hussey, Barnes-Holmes & Booth, 2016b; Leech et al., 2016; Nicholson & Barnes-Holmes, 2012), and *depression* (e.g., Hussey & Barnes-Holmes, 2012). In one article including the creator of the test as co-author (De Houwer et al., 2013), it was argued that implicit tests would be best approached as measures of *stimulus evaluations*. In that article, the authors argued that an evaluation could be defined in a nonmentalistic way in terms of the effect of stimuli on “evaluative responses.” Notwithstanding the fact that this conceptualization adds another possible construct to the long list of those that form the focus of published IRAP studies, we would argue that the best way to produce a functional understanding of precisely what is measured by the task will not be found in further theorizing (e.g., Barnes-Holmes et al., 2020a; Finn et al., 2018), but in properly controlled laboratory analyses of behavior within the task.

## A Behavior-Analytic Approach

As we hope to illustrate, from a behavioral perspective, the IAT (and IRAP) is *prima facia* a measure of the relative “strengths” of various stimulus relations, in the sense that it measures the relative ease with which functional response classes can be established when these are either consistent or inconsistent with prior learning (Roche et al., 2005). We are aware that, at least until recently, the concept of relation “strength” or stimulus relatedness was relatively novel in our field. However, several researchers have attempted to functionally define this concept. For instance, differences in stimulus equivalence yields following test probes for emergent relations of different nodal distance can be understood in terms of differences in stimulus relatedness (e.g., Moss-Lourenco & Fields, 2011), as can differences in the probability of the transfer of response functions (Fields et al., 1995; see also Arntzen et al., 2016; Fields, 2015; Fields et al., 2012; Mizael et al., 2016). Probabilities of derived relation yield have also been manipulated using overtraining in baseline conditional discriminations designed to lead to their emergence (e.g., Bortoloti et al., 2013). The “strength” of a stimulus relation, or the relatedness of stimuli within a relation, can also be conceptualized in terms of its resistance to change given competing reinforcement contingencies; also referred to by Tyndall et al. (2009) as class “stickiness.” Although the emphases of these conceptualizations differ, the outcomes they refer to are synonymous (i.e., the probability of functional or equivalence class emergence). It is also worth noting that it has long been a stated goal of stimulus equivalence researchers to develop a measure of stimulus relatedness as a function of training procedures (see Bentall et al., 1999; Bortoloti & de Rose, 2009; Doughty et al., 2014; Moss-Lourenco & Fields, 2011; Sidman et al., 1985).

Given the foregoing, imagine an individual with a long history of responding to flowers and bug stimulus exemplars as verbally equivalent to positive and negative evaluative terms, respectively. In colloquial terms, the individual *likes* flowers and *dislikes* bugs. Stated in a more technical way, the verbal and nonverbal response functions of positive and negative evaluative terms have also been established for flowers and bugs, respectively. This individual is likely to demonstrate response differences across IAT blocks that are differently configured as “consistent” and “inconsistent.” In other words, the IAT arguably measures the degree to which the establishment of functional response classes in the laboratory is facilitated or impeded by the existence of previously established functional or equivalence classes involving the relevant stimuli. Such a simple description of the IAT process avoids appeal to mentalistic concepts and instead focuses its analysis on the learning history of the participant completing the task.

Although latent variables as causal entities are not appealed to within behavior analysis, this does not mean that concepts like implicit bias or attitudes are not amenable to study from a behavioral perspective. For instance, De Houwer (2019) recently argued that implicit bias (or “attitudes”) may be in fact reconceptualized as instances of behavior *qua* behavior, without much cost to the cognitive perspective. To this end, implicit biases may be defined as “behavior that is influenced in an implicit manner.” In other words, during implicit measures, the sources of behavioral control are not easily discriminable by the test-taker. Such a conceptualization does not necessarily require a retreat to mentalism. However, De Houwer (2019) argued that defining implicit bias as behavior may also offer benefits to cognitive psychologists by allowing for clarity between the to-be-explained phenomenon and the explanatory accounts of that phenomenon. In effect, so long as the nature of the “bias” being analyzed is understood at the behavioral level, the behavior analyst can utilize and benefit from the same tools used by the social cognitivist.

A seminal study conducted by Watt et al. (1991) was probably the first to provide promise of a behavior-analytic methodology for assessing socially established verbal relations, which in turn whetted the palette of behavior-analysts to consider the experimental study of “attitudes” (see Roche et al., 2002). Watt et al. (1991) capitalized upon the stimulus equivalence phenomenon to examine how a sectarian social learning history in Northern Ireland in the 1990s might interfere with the emergence of new, incongruous stimulus relations. In particular, they attempted to establish two 3-member derived equivalence relations using a matching-to-sample (MTS) procedure, with the predicted equivalence classes containing a nonsense word, a Catholic name, and a Protestant symbol (class 1) and a nonsense word, Protestant name, and a Catholic symbol (class 2). The configuration of both classes ran counter to verbal histories of learning in Northern Ireland at the time, wherein Protestant and Catholic names and symbols were usually exclusive rather than equivalent. The study assessed Catholics and Protestant participants from two countries: Northern Ireland and England. Equivalence classes emerged reliably only for the English participants, who were not socialized within the sectarian culture of Northern Ireland of the late 1980s. Researchers interested in stimulus equivalence were highly excited about this finding, and spoke of it as providing the foundation for a discreet and perhaps more reliable behavior-analytic test of verbal histories of learning than direct questioning (e.g., Kohlenberg et al., 1993; Leslie et al., 1993; Merwin & Wilson, 2005; Roche & Barnes, 1996).

Grey and Barnes (1996) explicitly attempted to provide a definition of the concept of “attitude” from a behavioral perspective and used the stimulus equivalence paradigm as the first port of call for assessing attitudes defined in their terms. They drew upon the process of transfer of function (Barnes & Keenan, 1993) in describing how words within verbal classes acquire affective functions that produce responses that might parallel an attitudinal response of preference or disfavor. For example, if one member of a particular ethnic group is associated directly with aversive stimuli, or directly trained relations are established in language between a small number of exemplars of that class and aversive stimuli (e.g., “Catholics are lazy”), it would be expected that other members of the verbal class might acquire some of the response functions of that aversive stimulus. An attitude, therefore, might be conceived as a generalized affective response to a verbal class of stimuli (i.e., an equivalence class).

Grey and Barnes (1996) tested this idea in an experiment designed to establish three 3-member equivalence classes using an MTS procedure (i.e., A1-B1-C1, A2-B2-C2, A3-B3-C3) where all stimuli were nonsense syllables. The movie contents of video cassette tapes, labelled with A1 (sexually themed) or A2 (religiously themed), were shown to participants. Given the prevailing religious views at the time this study was conducted, and the sexual modesty these views promoted, evaluations toward religiosity and sexuality were expected to be positive and negative, respectively. After watching the A1 and A2 videotapes, participants were asked to categorize four more cassette tapes (labelled B1, C1, B2, C2) as either “good” or “bad,” without watching the content. In line with the transfer of functions effect, the video tapes were categorized in accordance with the relevant stimulus equivalence classes to which the original A1 and A2 video tapes belonged. In effect, the researchers had provided a primitive model of “attitudes” in terms of derived generalized evaluative responses. It is important to note that the researchers also showed that apparent attitudes change as a function of the context in which the relevant stimuli are presented. In particular, they found that when a sexual stimulus was presented alongside a “worse” violently sexual stimulus (a video tape containing offensive sexual activity), the former became more acceptable and was sometimes categorized as good, in comparison to the novel stimulus. This finding provided some nuance to the embryonic behavioral approach to attitudes and aligns with contemporary views in cognitive psychology that attitudes must always be understood contextually (e.g., Castelli & Tomelleri, 2008; Jost, 2019).

In another study, Roche and Barnes (1997) examined resistance to change in stimulus relations established through different means prior to efforts to establish incompatible stimulus relations. The researchers established sexual functions for nonsense word stimuli A1 and C1 and nonsexual functions for A2-C2 by pairing their brief presentation on a screen with sexual and nonsexual film clips, as appropriate. The establishment of the functional response classes A1-C1 and A2-C2, following the respondent conditioning procedure, was then tested with a simple matching test. In due course, the researchers attempted to reorganize the functional A1-C1/A2-C2 stimulus classes by exposing participants to a stimulus equivalence training procedure designed to produce the equivalence classes A1-B1-C2 and A2-B2-C1. However, performances on the equivalence test corresponded with the respondent conditioning, showing resistance to change towards current training and testing contingencies. It is important to note, however, for participants that did not pass the matching test following respondent conditioning, the laboratory programmed equivalence relations emerged more easily.

In an often-overlooked study, Plaud (1995) examined how aversive stimulus functions shared by members of a class might interfere with the formation of arbitrary stimulus equivalence relations consisting of subsets of that class. A within-subjects approach was employed, so that each participant’s performance in training and testing designed to lead to the formation of two stimulus equivalence classes, both consisting of images of snakes, was compared to their performance on an identical task involving flower images. Participants also filled out a fear of snakes questionnaire. Results showed that a higher reported fear of snakes was associated with requiring more blocks of training trials to reach criterion for equivalence class formation in the snake condition compared to the flower condition. It appeared reasonable to conclude, therefore, that the fear functions of the snake stimuli employed in the equivalence training procedure was the source of the delayed emergence of equivalence. However, other researchers suggested an alternative explanation.

Tyndall et al. (2004) assessed the “Plaud effect” more closely, suspecting that the effect was not due to the aversiveness of the stimuli per se, but rather to their shared functions and the relatedness of stimuli within the class (i.e., class “stickiness”). In their study, two functional classes of stimuli were established consisting of six S+ stimuli (responding towards was reinforced) and six S- stimuli (responding away from was reinforced). Two 3-member stimulus classes were then trained using an MTS procedure. One of four S+/S- stimulus combinations were trained across each of five conditions (S+ only, S- only, S+/S- one approach and one avoid class, S+/S- functions mixed within class, and a no-function condition). It was found that the formation of two 3-member distinct stimulus classes using 6 S+ stimuli (i.e., stimuli with same functions) required the most training trials. The quickest class formation was observed when stimulus equivalence classes corresponded with distinct functional response classes. These findings helped to identify features of learning contexts which impacted upon the acceleration and inhibition of stimulus equivalence class emergence. However, the manipulations across conditions in this study also inadvertently produced a methodology highly reminiscent of a procedure none other than the IAT. In other words, a first “behavioral IAT” could have consisted of comparing the rate of acquisition of two different stimulus equivalence classes containing real-world stimuli. It would not have required prior training with arbitrary stimuli and yet would still have allowed researchers to identify the configuration of socially established stimulus relations.

In a pivotal experiment, which offered a critical process-level analysis of class formation and change, Hall et al. (2003) established laboratory-controlled stimulus relations involving shapes and colors. On a computer screen, a color stimulus directly followed the presentation of a shape stimulus (i.e., shape A would be followed by red and shape B by green). The next stage involved establishing a directional response to the shape stimuli from stage 1. That is, when shape A was presented, a left positional keyboard press was reinforced. A right positional response was reinforced when shape B was presented. The final test stage of the experiment required participants to respond positionally to the colors from stage 1. The sample was split into two groups; consistent and inconsistent. That is, for the consistent group, contingencies for correct responding were consistent with training, whereas for the inconsistent group they were not. A higher percentage of correct responses was recorded for the consistent group. The inconsistent group responded at chance levels. Indeed, Hall et al. (2003) explicitly acknowledged that the effects seen in their study likely paralleled those observed in the IAT.

Roche et al. (2005) suggested that IAT effects could be understood in terms of differences in fluency of responding to different verbal stimulus class configurations. Roche et al. focused on the rate of *acquisition* of fluent responding to these different configurations. A lack of fluency in the acquisition of a specific configuration of response classes might be indicative of a previously established high rate of fluency in responding to the relevant stimuli according to the opposite pattern. Because such flexibility is established within a social context (i.e., the extent to which words can have multiple meanings and be categorized in different ways), Roche et al. concluded that IAT effects could be understood in terms of stimulus class configuration (in)compatibilities. The authors provided preliminary data to support this position, but this model was more rigorously tested by Gavin et al. (2008). Those researchers administered a training procedure designed to generate two 3-member equivalence relations using nonsense words as stimuli (i.e., A1-B1-C1, A2-B2-C2). A-B and B-C relations were directly trained whereas derived A-C relations were subsequently probed for in an MTS equivalence test. The idea was to administer a bare-bones IAT-type test following such training to assess whether it would be sensitive to the trained stimulus class configurations.

The bare-bones test administered by the authors embraced several of the conceptual and methodological concerns outlined earlier. In particular, corrective feedback followed all (not just incorrect) responses. Response windows were limited to 3,000 ms and missed responses (i.e., over 3,000 ms) were classified as incorrect. The rationale here was that the presence of the response window led to more errors under whichever set of contingencies such errors were in principle more likely (cf. Bolsinova & Maris 2016). In other words, the idea was to bring error rates under stimulus control directly within the procedure. Thus, response time was recorded as the time from trial onset until first emitted response. However, in this study (but not later studies) the usual IAT/IRAP on-screen instructions describing the reinforcement contingencies for each block were present during each trial. The primary test score was calculated in terms of a difference in percentage *response accuracy* across the two test blocks, rather than in terms of a time-based IAT-*D* score.

In the consistent block of the modified IAT (that is, the block in which response contingencies were consistent with the trained relations), a common positional response on the keyboard (e.g., “D” key) upon the presentation of A1 and C1, and a different common positional response (e.g., “K” key) upon the presentation of A2 and C2 stimuli, was reinforced. In the inconsistent block, A1 and C2 required a common response whereas A2 and C1 required an alternative common response for reinforcement. In effect, the functional response classes established in the inconsistent block of the modified IAT were incompatible with the equivalence relations established in phase 1. The test proved sensitive to the training history insofar as higher accuracy was recorded on the consistent block. This provided the first evidence that an explanation of IAT effects in terms of stimulus relation compatibilities was sufficient.

Later, Ridgeway et al. (2010) replicated this general effect. The authors went on to expose participants to MTS training designed to reorganize the previously established equivalence classes. These participants were subsequently reexposed to the modified IAT. The performances on this second IAT reflected the modified equivalence relations based on response accuracy, but curiously not based on response times (although this was not highlighted in the article). In other words, response accuracies proved to be a more sensitive measure of contingency change than response latency (it is interesting that this is consistent with recent findings in other measures; cf. Cummins & De Houwer, 2022). Several other studies then followed, employing a modified IAT to assess stimulus relations that had been established in the natural environment (e.g., sexual stimulus classes; see Gavin et al., 2012; Roche et al., 2012).

Further modifications to the IAT procedure, involving a more interpretable scoring metric (discussed later) and the removal of unnecessary methodological features of the IAT (e.g., persistent on-screen instructions), seemed to eventually justify a new name for this procedure that reflected its behavior-analytic orientation. The name chosen directly described the process that appeared to underlie the basic effect, differences in the speed of acquisition of functional response classes under different reinforcement contingencies. Thus, a new test format including additional features outlined below, was named the *function acquisition speed test* (FAST; as a convenient acronym and a nod to the speed of administration).

## The Function Acquisition Speed Test (FAST)

The FAST, like the IAT, is a computer-based test used to measure stimulus relatedness, the strength of which might be used with caution as a proxy for attitudes understood, in turn functionally as a network of related stimuli (Grey & Barnes, 1996; see also Roche et al., 2002). The measure has undergone several adjustments following its first introduction by O’Reilly et al. (2012), and these will be examined in more detail below. However, in its current state, the FAST consists of two blocks that are presented in a random order. The response contingencies on one block are consistent with the learning history of the participant (i.e., the “consistent” block), whereas those during the “inconsistent block” are inconsistent with that history. Both blocks typically contain 50 trials, which in turn involve the presentation of a single stimulus at the center of an otherwise blank screen. The on-screen stimulus is an exemplar from one of four verbal categories: an attribute stimulus (of which there are two, usually positive and negative words), or a category stimulus (of which there are also two, for example, male, and female words, traits or labels). The participant is informed prior to the beginning of the block that they must respond to the stimuli within 3,000 ms, using one of the two designated keys on their keyboard (e.g., a “left” and “right” key is normally assigned, using the “Z” and “M” keys, respectively, as an affectionate nod to a long tradition with derived relational research). Negative feedback is delivered for incorrect, or indeed, missed responses (e.g., “WRONG” in red on screen for 500 ms), and correct responses result in positive feedback (e.g., “CORRECT” being presented on screen for 500 ms). The pretest instructions also inform the participant that they must learn to respond correctly on the basis of feedback provided to them after each response. As in the IAT, in the consistent block, items from compatible stimulus categories share a response key requirement whereas items from incompatible classes share distinct response requirements. During the inconsistent block stimulus exemplars from compatible response categories must be responded to using different response keys, whereas stimulus exemplars from incompatible categories must be responded to using the same response key. Instructions are presented in the short interval between blocks to inform participants that the response contingencies may have changed but no instructions appear on the screen at any stage during tasks and no instructions are ever given as to how to respond appropriately.

The FAST procedure deviates significantly from the IAT and IRAP in sometimes apparently minor topographical ways, but in ways that matter considerably from a functional perspective. As we outline below, the FAST has evolved to increasingly align with a functional approach to the analysis of behavior as additional features are analyzed in both in-house and in published studies. What follows is a review of the short history of the FAST research program, detailing the evolution of various methodological features.

The novel FAST method was the natural product of published studies on (1) the acquisition of stimulus equivalence and functional response classes; (2) the backward engineering of the IAT; and (3) published modifications to and conceptual analyses of the resulting procedure. Early iterations of the test paralleled the single-category variant of the IAT (e.g., Karpinski & Steinman, 2006). That is, the first explicitly named FAST study, O’Reilly et al., (2012) explored the utility of assessing the speed of acquisition of functional response classes, for the purpose of indexing the strength of relations within *a single class only*. In particular, after establishing two simple zero-node two-member arbitrary relations involving nonsense words as stimuli, only one of these classes was targeted for indexing in terms of stimulus relatedness. The test involved instating reinforcement contingencies in the consistent block that required common responses to both members of a single class, and a second common response to two novel stimuli *not involved in prior training.* The inconsistent block involved establishing a common response for one member of an established class and another novel stimulus, and a second positional response for the other member of the established class and yet a further novel stimulus. The idea was that such a procedure might provide a “pure” index of individual relation strength, *not relative to* the strength of relations already established among members of a second stimulus class. The procedure was generally effective, successfully generating differences in class acquisition rates (measured as the number of trials required to produce 10 consecutive correct responses) across the two test blocks. A further study (O’Reilly et al., 2013), extended the effect to assess the strength of relation amongst stimuli within 1-node derived relations. In these first two studies the block order was counterbalanced with an abundance of caution. However, data inspections showed that the very small block order effects being controlled for with counterbalancing were eliminated just as effectively by block order *randomization*. Thus, in all iterations of the test going forward block orders were randomized rather than counterbalanced.

Although the idea of an absolute, single-target test was initially the goal, in-house research quickly indicated that FAST effects were generally stronger when two classes were being assessed simultaneously. It was reasoned that, using a relativistic (i.e., double target) procedure rather than an absolute one, allowed the functional response classes being established to be accelerated by the already existing behavioral momentum (Nevin & Grace, 2000) relating to two separate relations simultaneously (i.e., responses were controlled by S+ and S- control simultaneously rather than only one form of control at a time). Likewise, during an inconsistent block, both functional response classes would be incompatible with two established classes, rather than just one. A similar conclusion was reached within the IAT literature but for different reasons (e.g., Robinson et al., 2005). Thus, a relativistic approach was adopted in the FAST going forward. In hindsight, an “absolute” measure of relatedness is inherently at odds with the contextualistic perspective of stimulus relations research, as others also concluded (see, for example, Hussey et al., 2016a). In particular, it is not reasonable to expect that any stimulus has a fixed response possibility in the presence of another conditional stimulus; that probability is moderated by the response options available (or lack thereof), and none represent the “true” measure of intraclass stimulus relatedness.

In addition to the methodological changes that led to the development of the FAST, changes to the scoring method were also made. That is, rather than use raw response accuracy differences across test blocks as the metric of stimulus relatedness, the FAST began to consider the use of a metric that would have more face validity as a measure of the rapidity of the acquisition of a functional response class. The simple idea was that the number of tasks presented per block should be potentially infinite, and blocks should continue until the participant reached a response criterion in terms of a particular number of successive correct responses. That way, the rapidity of class acquisition would be measured in terms of the number of trials required to reach criterion rather than the number of trials correct on a finite block. A test score could then simply involve calculating the trial requirement differential across blocks.

The introduction of the trials-to-criterion method was accompanied by the introduction of two single short baseline blocks (as opposed to practice blocks), one before and one after the two key blocks, and both involving different arbitrarily chosen nonsense words unrelated to the rest of the test. The rationale was that these would provide a baseline functional response class acquisition rate for that individual participant. A mean acquisition rate for these baseline blocks could be used to moderate the acquisition rate differential across the two key blocks. In other words, it would facilitate idiographic style standardization that would correct raw response class acquisition rate differentials by the baseline rate of acquisition. To reflect these changes, a novel, fluency-based scoring metric that combined speed and accuracy, called the strength of relation (SoR) index was introduced (O’Reilly et al., 2012). The first iteration involved dividing the trials to criterion differential across blocks by the mean trial requirement on the baseline blocks for each participant. In the second iteration of the index (O’Reilly et al., 2013), the denominator was the natural logarithm of the mean baseline block trial requirement. One study conducted using the latter method (Cummins et al., 2019) used the FAST to measure the impact of behavior-change focused health education interventions. Participants were health workers assigned to Positive or Negative messages regarding the use of condoms as disease prophylactics, or to a control (no message) condition. All participants then completed a FAST designed to assess relations between condoms and positive and negative evaluative stimuli. Results showed that the FAST was sensitive to the content of these brief messages. That is, the performances of positive message condition participants indicated stronger relations between condoms and positive evaluative terms relative to negative. This pattern was reversed for the negative message group. These results supported the idea that the FAST method was sensitive to verbal relations organized in a brief and naturalistic intervention.

Despite promising results for the native FAST, there were two shortcomings with this SoR scoring metric and associated baseline blocks. Firstly, after dozens of in-house experiments, it was concluded that baseline blocks showed the slowest overall acquisition rates; they were not generally slower than consistent and faster than inconsistent blocks, as initially anticipated. Having replicated this effect in-house with several different stimulus sets, it appeared that the novelty of the stimuli alone was the source of the slow acquisition rates during baseline blocks. Indeed, previous studies had found that the level of familiarity of stimuli (Holth & Arntzen, 1998), as well as the presence of salient emotive or conative stimulus functions for stimuli (Arntzen et al., 2018) is associated with an accelerated rate of stimulus class formation and reorganization. Thus, the use of baseline training blocks was abandoned. Second, the trials-to-criterion component of the SoR index was problematic in its crudeness. For example, a single error on the 10th trial following a run of nine correct responses required the participant to be exposed to at least another 10 trials to satisfy the usually 10 correct successive responses acquisition criterion. This caused enormous variations in response requirement criteria across blocks and across participants. In other words, the measure was inherently noisy.

A finer metric was conceived, in which the rate of learning on each block was calculated in terms of the angle of the regression line of the learning curve. The learning curve itself was produced using a cumulative record-style plotting system in which correct responses on the Y axis were plotted against time on the X axis. Correct responses produced standard increments on the Y axis over continuous time on the X axis, including a 500 ms response feedback period during which the operanda were disabled (i.e., responding was not possible). The FAST score was then calculated as the slope (of the regression line) of the consistent block learning curve minus the slope (of the regression line) of the inconsistent block learning curve. It was literally and transparently a learning rate differential score with very appealing face validity. Such a method allowed for a more sensitive analysis of moment-to-moment change than the previous SoR index, and a better functional understanding of the dynamics of the test performance. It would also allow for analyses of the trial-by-trial task performance dynamics, which was used to facilitate research into what constituted the ideal block length, which themselves had to be of a fixed length to calculate the score. For example, in one study examining the strength of preexisting verbal relations characteristic of gender stereotypes (Cartwright et al., 2016), it became evident that learning rates typically continue to differentiate as trials progressed through each of the blocks. The dynamics of the performance displayed in the moment-to-moment data corresponded with that of dozens of in-house experiments that showed that learning rates do not differentiate well across blocks within the first 10 trials or so, and differentiation in learning trajectories across blocks appears to begin to plateau after 50 trials or so. Thus, although that research is unpublished, a block length of 50 trials was hit upon and appears to have served well in the interim.

Practice blocks were also considered and tested in dozens of in-house studies, but they made little difference to the outcome of the FAST in terms of stabilizing response accuracy or speed on the subsequent critical task blocks. It is important however, that the reader understand that the FAST is conceived as an acquisition rate test, and so providing practice might confound the very variable of interest. That is, practice will serve the purpose of creating a steady state behavior, as is achieved in the IAT and IRAP, before response speed or fluency differences are assessed across the critical test blocks. However, within the behavioral tradition, behavioral variability is our very subject matter of interest (cf. Sidman, 1960; Skinner, 1976). Therefore, if the contingency shifts across task blocks are indeed the source of differences in performance across blocks, then this should be visible during acquisition itself, albeit with some noise. In other words, in both the IAT and IRAP, the very phenomenon of interest to behavior analysts (i.e., behavior *qua* behavior) is being obfuscated through repeated practice before behavioral metrics are taken. Indeed, in both measures, criteria are applied during practice to screen and eliminate participants who do not show such steady state behavior (Barnes-Holmes et al., 2010a; Hussey et al., 2015). Thus, practice obscures the dynamics of the behavioral performance in which we should be interested, even if it does achieve the purpose of eliminating a degree of noise in the data for statistical inference purposes. Of course, we must strike a balance between limiting one source of noise in the task that is not of interest (i.e., random variance) while also capturing another source of noise which *is* of interest (i.e., systematic variance). Striking this balance remains an issue, but contemporary FAST studies generally omit practice blocks.

One of the most critical aspects of the behavioral account of implicit measures (and an aspect that is often assumed even in cognitive accounts) is the prediction that the magnitude of effects in implicit measures should be proportionate to the relatedness of the probed stimuli. In our field, research into stimulus equivalence yields has established that yield is functionally related to the fluency of the relevant baseline relations (e.g., Bortoloti et al., 2013; Fields et al., 1995). Corresponding to this, FAST scores should theoretically increase in tandem with increasing stimulus relatedness. Although the same assumption has been made by IAT (and IRAP) researchers, however, this assumption has to our knowledge never been tested empirically and directly in laboratory-controlled research despite an impressive literature base in both cases.

Fortunately, addressing this issue is surprisingly easy because relatedness can be conveniently objectively manipulated by overtraining (Bortoloti et al., 2013) or assessing relations of differing nodal distance (Moss-Lourenco & Fields, 2011). Cummins et al. (2018) and Cummins and Roche (2020) used both methods to assess the impact of controlled relatedness on FAST scores. The 2018 study involved administering MTS training designed to establish stimulus equivalence relations across different periods of time and with different numbers of iterations across experimental conditions. The study also involved a control condition in which participants were exposed to a FAST consisting of stimuli that had not been presented during any prior phase, and a second control condition involving the FAST assessment of real word associations of standardized strengths based on the South Florida norms index (Nelson et al., 1998). In all conditions, except for the real word condition, stimuli consisted of nonsense syllables. The conditions involving training of arbitrary stimulus relations consisted of either one MTS session, two MTS sessions spread across 1 week, three MTS sessions spread across 2 weeks, or three MTS sessions all conducted in one sitting. A FAST to assess the strength of relations within and between the established equivalence relations was administered following the final sessions of each of these four training conditions.

FAST scores increased as a function of controlled stimulus relatedness, using the slope scoring method (Cartwright et al., 2016). It is interesting that the real word condition produced the strongest effects in terms of learning rate differentials, with the differential effect attributable to both a degree of facilitated learning on the consistent block and impeded learning on the inconsistent block. This was the first evidence produced that scores on *any* implicit-type test could be understood to be a function of the fluency of relational responding with respect to the stimuli used in the test. In addition, it provided important information that even overtrained laboratory relations do not have the fluency of real-world verbal relations; thereby providing us with reference points for interpreting test scores (as opposed to the beginnings of standardization of test scores).

In a follow-up study, Cummins and Roche (2020) investigated the impact of varying nodal distances on FAST scores. Two 4-member equivalence classes (A1-B1-C1-D1, A2-B2-C2-D2) were established using an MTS procedure involving training each zero-node pair to criterion in succession (e.g., first A1-B1 and A2-B2, then B1-C1 and B2-C2). It is important to note that derived relations were not tested at this point. Three FAST tests were then administered to all participants in a counterbalanced order. The first was a zero-node FAST, in which the strength of A-B relations were tested. A 1-node FAST then probed for derived A-C relations, while the final two-node FAST probed for A-D relations. A MTS test for all derived relations was then administered. At the group level, FAST scores decreased as nodal distance increased as expected.

It is interesting that the block slope score for the inconsistent block significantly increased as a function of increasing nodal distance, whereas slope scores for the consistent block remained unaffected. These trends were visible at the group level and for most individual participants, although a large amount of variability in individual scores was also observed (and as we discuss in the next section, this method of individual-level score interpretation was rather crude in hindsight). This move away from group-level analyses and towards an individual-level of analysis represents the most pressing next step for research using the FAST. Of course, individual variability is commonly seen on tests for derived relations, especially across differing assessment methods (e.g., Bentall et al., 1999). This alone, however, should not be grounds for a retreat to exclusively group-level analyses at the expense of individual-level analysis. Indeed, other implicit measures also exhibit a substantial degree of variability at the individual level (e.g., Klein, 2020; Hussey, 2020).

The foregoing outline of the evolution of the FAST methodology provides a case study in how to develop such a test inductively from the ground up based on well-understood behavioral principles. It might be argued, however, that no harm is done by presenting a test to the public before it is perfect, and that in this regard tests like the IRAP have done a service to the community by making themselves available for investigation by the research community. Indeed, within the contextual behavioral science field such strategies are often described positively as “progressive.” However, it is important to be clear on the line between progressiveness and recklessness. The reckless promotion of a methodology (and corresponding claims about its utility and interpretability) in the absence of reproducibility and sufficient empirical grounding can waste the time of researchers for years and misdirect research energy. This point was recently made anecdotally in a widely read and discussed blog by one member of the behavior-analytic community regarding the IRAP (Drake, 2022).

## Future Research Directions

Starting from equivalence training based methods in the tradition of Watt et al. (1991; e.g., Tyndall et al., 2004, 2009), to modified implicit association tests (e.g., Gavin et al., 2008), to a native FAST (O’Reilly et al., 2012) and its most recent incarnation (e.g., Cummins & Roche, 2020), the FAST has been developed generally on the basis of laboratory-based studies using experimentally controlled stimulus relations to examine the properties of test performances in a depth greater than typically seen in implicit measures research. However, many empirical questions remain outstanding. For example, a deeper understanding of the relationship between enforced response times windows (limited hold parameters) and response fluency has yet to be conducted. The relationship between these two variables is almost certainly complex, and the effect of response windows on response fluency is likely to differ at different points in the trajectory of learning.

Another issue yet to be explored relates to the reinforcement contingencies used in these tests. In particular, a systematic analysis is required of simulated tests in which feedback is provided for correct responses only, or incorrect responses only, alongside an examination of the effect of a thinning of the reinforcement schedule on test scores. It may well be that a thinning of the schedule reduces the fluency on both blocks, or does so disproportionately across blocks, thereby enhancing the sensitivity of the contingencies to preexperimental learning differences. Indeed, such an investigation could also encompass these same manipulations within the IAT procedure to gain a more detailed understanding of their impact across different procedures.

Yet another question relates to the optimal scoring metric for the test. For instance, rather than assess response fluency differentials across two single blocks of the test, an overtraining approach could be taken in which the change in the fluency differential across blocks is accessed across multiple iterations of the test. Larger effects on the first iteration should persist across more iterations of the test than will weaker effects. Thus, a novel and more reliable metric of stimulus relatedness, might involve identifying the point at which learning rate differentials across blocks approach zero, or reach a half-way point between the differential on the first iteration and a zero-point differential (i.e., a half-life index). Further questions also remain regarding the optimal number of trials per block, the potential use of various instructions, and acceptable data standardization methods (e.g., log transformation).

In this vein, the authors are currently exploring the utility of a new metric that deals with one potential confound of the learning slope differential method. In particular, this method does not protect against fortuitous sequences of correct responding produced by rapid random responding. Simulations can trivially demonstrate that a high rate of random responding will produce block-slope scores that are not differentiable from medium-speed highly accurate responding, although the latter is clearly under greater stimulus control than the former. It would ideal if learning rates would be corrected for by the attendant rate of incorrect responses per minute. A simple alternative, therefore, would be to calculate the difference between correct and incorrect responses per minute for each block, resulting in a fluency score for each block that reflects the proportionate rate of correct to incorrect responding. The overall FAST effect could then be calculated by subtracting the fluency score for the inconsistent block from that observed for the consistent block, producing what we might call a rate fluency differential score (RFD). It should be noted that this metric gives primacy to accuracy (fast and inaccurate responses will result in very low scores compared to slow and accurate responses), although still accommodating response times after accuracy has been maximized.

It is important to note at this point that discussions regarding “optimal” scoring methods, optimal number of trials, and so on, are bound to lead nowhere without consideration of the reasons for why the measure is being used in each context. Just as there is no “true” relatedness of a stimulus class, there is no “true” optimal permutation of this measure (or indeed, any measure). Identifying that which is most optimal for the measure must always be done with reference to clear and specific criteria (e.g., improved individual-level precision, improved group-level psychometric properties, improved correlations with varying degrees of experimentally manipulated stimulus relatedness). These different criteria may well be best optimized by different procedural variations. It is critical for future work developing the FAST to make clear what specific criteria are attempting to be optimized with these variations; this in turn will also provide a more in-depth understanding of the nature and dynamics of the measure.

Recent developments in relational density theory (RDT; Belisle & Dixon, 2020a) have raised the opportunity for cross fertilization of ideas regarding the assessment of stimulus relatedness. In particular, RDT employs the Newtonian concepts of density, volume, and mass to characterize response probabilities within and between stimulus relations in larger networks. The theory is designed to make sense of nonlinearity of equivalence responding where relations compete within a network and where individual stimuli may control behavior more or less effectively as a result of variances in such variables as familiarity or novelty (see Belisle & Dixon, 2020b). It is interesting that the developers of this account also explicitly draw on the concepts of momentum and resistance to change to explain why current contingencies have an impact on current relational responding. This overlap in conceptualization represents an opportunity for the FAST procedure to be used as a quick and easy method by which relational networks can be mapped in terms of the relatedness between stimuli and their resistance to change. To this extent, RDT and the FAST would appear to be complementary in terms of their use of Newtonian metaphors for understanding different behavioral probability under competing contingencies. Of course, multiple analyses would be required to assess the intricate dynamics of any one relational network, but complexity alone should not be a barrier to our efforts.

On a related note, the FAST may be our first empirically grounded tool that can be used to fulfill one of the ambitions of contextual behavioral scientists to capture verbal behavior in “free flight,” as envisaged by the late Williard Day. That is, variances in the acquisition or impairment of learning rates as a function of the compatibility of stimulus relations in which individual stimuli co-participate can now be measured somewhat reliably, and in principle the interacting forces at work in an individual relation response can be quantified. Of course, once again this is an incredibly complicated task, but it has not yet been achieved by any other method despite it being a rallying cry of the very purpose of RFT itself, out of which the IRAP method also emerged. The speed of administration of the FAST, in contrast to the cumbersomeness of the IRAP, lends itself more readily to this goal.

It might be suggested that the FAST method is also relevant to recent theoretical positions encapsulated within the differential arbitrarily applicable relational responding effects model (DAARRE; Finn et al., 2018), and more recently the multidimensional multilevel framework (MDML; Barnes-Holmes et al., 2020a; formerly the hyperdimensional multilevel [HDML]: Barnes-Holmes et al., 2020b). These models have sought to explicate various aspects of performance on the IRAP, including differential trial type effects and the level of relational development and complexity. It is important to understand, however, that these are theoretical models that provide interesting and potentially powerful rubrics with which to understand relational behavior. They are not, however, intrinsically testable, nor should research energy be devoted to attempting to test these models in a reversal of our usual ground-up approach to research. Insofar as these models provide a framework within which to understand behavior meaningfully, pragmatically, and parsimoniously, they may be of use to some researchers in some analytic contexts. However, it is important that no technical developments within our field are obliged to easily submit to explanation by these accounts. The reliability and validity of the FAST methodology, or any other methodology, should not stand or fall to any extent based on the degree to which it aligns or misaligns with these post-hoc theoretical models. The emergence of the FAST from systematic laboratory-controlled ground-up research should always take precedence over its alignment with a theoretical position. In this sense, we do not see it as particularly useful to explicate an account of how the findings of the FAST research outlined here fit with these models. We are more interested in seeing how proponents of these models fit them with the findings of FAST research studies.

As mentioned above, one important guiding principle for the future of FAST research is a stronger emphasis on individual-level analysis. Studies using the FAST to date have typically focused on the group-level of analysis (but see Cummins & Roche, 2020). Indeed, the same can be said for the IAT and IRAP (although see Finn, 2020). In effect, both the fields of social cognition and behavior analysis are top-heavy with examinations of these measures at the group-level, with comparably little individual-level analysis. Indeed, even in those few studies that have examined individual-level data, they are limited in that the precision of individually estimated scores is rather poor (Klein, 2020; Hussey, 2020). What is needed now for the FAST (and indeed, other measures) is a renewed focus on the individual-level of analysis and improvement of the estimation of individual-level scores. In particular, FAST researchers should seek to reduce unwanted random error variance while also more precisely estimating the systematic variance of interest (i.e., variance in scores due to stimulus relatedness). This is clearly a lofty challenge and there have been few clear guidelines on how to achieve this. One method would be to estimate and understand the (im)precision associated with measurement instruments such as the FAST. For every measurement procedure which produces a score (i.e., a single numeric value), there is necessarily statistical uncertainty around this value. Within contexts such as educational testing, this uncertainty is quantified by computing confidence intervals around the scores of individual participants. One common approach to doing this is by using the *standard error of measurement* (Se*m*; not to be confused with the standard error of the mean; Dudek, 1979). However, the SE*m* requires known values for the test instrument, such as the population standard deviation and the sample’s test reliability. An alternative approach extensively used in statistical analysis, and which has already been used with other implicit measures (Hussey, 2022; Klein, 2020), is a computational approach known as *bootstrapping* (cf. Hussey, 2022 for a detailed explanation).

Regardless of the computational method used, by estimating the uncertainty around scores in the FAST, this would allow researchers to make inferences about *individuals.* For instance, this method would allow us to determine whether an individual’s score is “significantly” greater than the null point (where significance can be tested based on the inclusion/exclusion of the null point within the individual’s confidence intervals), or whether two individuals differ significantly from one another. By having a direct metric of the FAST’s precision at the individual-level, researchers in turn can take steps to try to improve this precision (e.g., by increasing the number of trials used in the task) and gain better insight into the specifics of individual-level responding. Pursuing with this line of investigation regarding the meaning of individual participants scores both in terms of a functional understanding of the score itself and in terms of improving our confidence of the representativeness of that score for that participant using statistical methods will aid further in the FAST’s development as a truly behavior-analytic implicit measure.

Whatever the results of the interesting process-level research that will be conducted going forward, it is crucial to the aim of our research agenda, and in the interest of collegiality and openness within our science, that no methodological feature or scoring mechanism should ever be considered integral to the method, even where empirically supported. In other words, the FAST should be seen as a general methodological strategy linked to a very basic behavioral account of the core effect, in the same way in which applied behavior analysis represents a scientific strategy rather than a specific, restrictive approach to treatment. All and any methodological and metric variables should be open to modification without claims of the bastardization of the general method. It can be argued that measures such as the IRAP that have achieved apparent proprietary status, with rigid methodological features, instructions, and scoring methods may serve to stagnate research, particularly if results garnished with novel methodologies are considered inadmissible under the umbrella term of the original methodology. If methodological differences are substantiated by sound measurement properties, they should be embraced. Of course, such a wide umbrella approach to methodology can open doors for the possibility of p-hacking (wherein multiple criteria are employed in analysis until statistically significant results are found). However, the risk of this can be strongly mitigated by preregistration and open science practices, allowing researchers to make clear and transparent delineations between confirmatory and exploratory work (Nosek et al., 2020).

Going forward we also need to be mindful of errors not to be repeated, because they may have been engendered by the IRAP research strategy. That is, the IRAP literature base is currently vulnerable to criticism of unreliability and HARKing based on the repeated use of analytic practices which are far from best practice in psychological sciences. In particular, sample sizes in IRAP studies are typically very low with a median of 64 in 2022 (see Hussey, 2023). This may not seem like a particularly low sample size, but this issue needs to be placed in the context of the typical way in which IRAP data is analyzed. That is, IRAP studies tend to use large multifactorial ANOVAs (e.g., 4X2 [trial type X group] or 4X2X2 [trial type X group X block order]), as well as difference from zero *t*-tests for each trial type. This is sometimes in addition to multiple correlations between individual and combined block scores and explicit measure scores. Thus, a typical IRAP study could involve around a dozen statistical tests involving the re-use of data in multiple comparisons (e.g., two ANOVA main effects, multiple interaction effects, four difference from zero tests, and four correlations). In this case, a sample size of 64 leaves the analysis woefully underpowered. In addition, statistical correction is typically not applied for multiple pairwise comparisons on the grounds that these are “exploratory.” This unhealthy mix of study design and analytic method features inflates false positives to an unacceptable degree and renders the studies practically useless as exploratory studies, and questionable as confirmatory studies. In particular, Cramer et al. (2016) outlined how even for a 2X2 ANOVA the false positive rate is amplified beyond a 0.05 probability to 0.14 because of the increased opportunity for false positives on individual main effects and interactions. This simple effect is amplified further for more complex study designs. In effect, the complexity of the IRAP methodology, leading to the requirement for complex designs and complex scoring algorithms, compromises study results by a reduction in statistical power (low rate of true positive detection) and an increase in the false positive rate. This leads to low confidence in the research literature in terms of the replicability of results and to low confidence in theoretical models designed to explain the published data. It is clear that research needs to move in the opposite direction, involving simpler study designs that are highly powered and ideally involving preregistration, combined with a commitment to avoiding post-hoc comparisons, especially in the absence of proportionately controlled error rates.

## Conclusion

The aim of this article was to provide the research backstory for the function acquisition speed test, sketching its origins as a behavior-analytic implicit measure, its status in terms of experimental investigations into the task, and its (hopeful) future development as a more individual-level measure of stimulus relatedness. This was necessarily achieved by contrasting many of its features against those of the IAT and the IRAP. The FAST methodology is offered as a general starting point for indexing the strength of relations between stimuli within and across classes in a relatively indirect and convenient way. In that sense, its status is no different to that of a wide variety of equivalence class training and testing methods, such as matching-to-sample, card sorting, and a wide variety of fluency criteria applied during equivalence class training. These are merely the formats employed to harness well-understood behavioral processes and they are not themselves the process. As it stands, the methodology, at its current stage of evolution is public domain and open source (https://github.com/JamieCummins/fast-js), and decidedly not proprietary. There is no “official” version of the relevant software and there are no gatekeepers to comment on how variations might align with the version approved by its creators. It belongs to the scientific community. Although we are happy to share relevant software, we encourage researchers to (attempt to) replicate existing findings, explore new configurations of the procedure, and push the measure’s development forward both conceptually and methodologically.

At present, the FAST might be considered as part of the toolkit of researchers attempting to establish and assess derived relations. Other novel methods have been explored in recent years, including card sorting (Fields et al., 2014; Fields et al., 2012), although this indexes only the emergence or nonemergence of a whole class. Although useful, card sorting is not a nuanced measure. In contrast, the advantage of the FAST method is that it can be administered more than once during the equivalence training protocol and will provide a measure of the increase in relatedness of stimuli within the class across time. It also allows for independent probing of symmetrical and transitive relations. We hope that this review and case study can serve as a touchstone for researchers getting acquainted with the FAST measure and implicit testing research more generally and provide a helpful springboard for those wishing to develop this type of procedure further, or indeed to develop their own novel procedure from first principles. To the extent that the current FAST method evolves and changes and is improved upon beyond recognition by other researchers, this contribution will have been a success.
